# Dr. Charles A. O’Dea

**Published:** 1918-05

**Authors:** 


**Dr. Charles A. O'Dea,** Erie Pa., Niagara University Medical Department, Buffalo, 1895, died March 9, age 43.

